# Interleukin 1 beta promoter polymorphism is associated with keratoconus in a Japanese population

**Published:** 2013-04-11

**Authors:** Takenori Mikami, Akira Meguro, Takeshi Teshigawara, Masaki Takeuchi, Riyo Uemoto, Tatsukata Kawagoe, Eiichi Nomura, Yuri Asukata, Misaki Ishioka, Miki Iwasaki, Kazumi Fukagawa, Kenji Konomi, Jun Shimazaki, Teruo Nishida, Nobuhisa Mizuki

**Affiliations:** 1 Department of Ophthalmology and Visual Science, Yokohama City University Graduate School of Medicine, Kanagawa, Japan; 2 Yokosuka Chuoh Eye Clinic, Kanagawa, Japan; 3 Ryogoku Eye Clinic, Tokyo, Japan; 4 Department of Ophthalmology, Tokyo Dental College, Ichikawa General Hospital, Chiba, Japan; 5 Department of Biomolecular Recognition and Ophthalmology, Yamaguchi University School of Medicine, Yamaguchi, Japan

## Abstract

**Purpose:**

Polymorphisms in the interleukin 1 alpha (*IL1A*) and *IL1B* gene regions were previously associated with keratoconus in a Korean population. In the present study, we investigated whether the *IL1A* and *IL1B* polymorphisms are associated with keratoconus in a Japanese population.

**Methods:**

A total of 169 Japanese patients with keratoconus and 390 Japanese healthy controls were recruited. We genotyped one *IL1A* single nucleotide polymorphism (SNP; rs2071376) and two *IL1B* SNPs (rs1143627 and rs16944) to compare the frequencies of alleles, genotypes, and haplotypes between cases and controls.

**Results:**

Statistically significant association was observed for rs1143627 (−31 T>C) in the *IL1B* promoter region; the T allele of rs1143627 was associated with an increased risk of keratoconus (p=0.014, corrected p value [pc]=0.043, odds ratio=1.38). The C allele of rs16944 (−511 C>T) in the *IL1B* promoter region had a 1.33-fold increased risk of keratoconus, although this increase did not reach statistical significance (p=0.033, pc=0.098). The TT genotype of rs1143627 was weakly associated with an increased risk of keratoconus (p=0.033, pc=0.099, odds ratio=1.52). However, no significant differences were found in the allele and genotype frequencies between the cases and controls for rs2071376 in *IL1A*. Regarding haplotypic diversity, the haplotype created by the T allele of rs1143627 and C allele of rs16944 was associated with a 1.72-fold increased risk of keratoconus (p=4.0×10^−5^, pc=1.6×10^−4^).

**Conclusions:**

Our results replicate associations reported recently in a Korean population. Thus, *IL1B* may play an important role in the development of keratoconus through genetic polymorphisms.

## Introduction

Keratoconus (OMIM 148300) is a non-inflammatory corneal disorder characterized by progressive thinning of the corneal tissue, which can lead to severe visual impairment. Prevalence rates for keratoconus vary widely across geographic areas and studies, ranging from 0.0002% to 2.34% [1]. The exact etiology of keratoconus remains uncertain, but the disease is currently thought to be triggered by various genetic, as well as environmental, factors. Family and twin studies provide convincing evidence of the importance of genetic factors in the development of keratoconus. The prevalence of keratoconus in relatives of keratoconus cases is greater than that of the general population [2-4], and monozygotic twins show a high concordance rate for keratoconus [5-7].

To date, many genetic loci associated with keratoconus have been reported with linkage analysis: 1p36.23–36.21, 2p24, 3p14-q13, 4q, 5q14-q21, 5q32-q33, 8q13.1-q21.11, 9q, 12p, 13q32, 14q11.2, 14q24.3, 15q22.33-q24.2, 16q22.3-q23.1, 20p11.21, and 20q12 [8,9]. Of these loci, 20p11.21 harbors the visual system homeobox 1 (*VSX1*) gene. Several studies have identified mutations in *VSX1* as a cause of keratoconus development in multiple ethnic populations [9-17], and *VSX1* could be a strong candidate gene for keratoconus. However, a similar number of studies did not find *VSX1* mutations to be specific to the disease [18-26]; therefore, *VSX1* mutations may be responsible for a small fraction of keratoconus cases, suggesting that other genetic factors have more powerful effects on the development of keratoconus. In addition to *VSX1*, several other candidate genes have been reported, including superoxide dismutase 1 [16,27,28], lipoxygenase [29], transforming growth factor, beta 1 [30], secreted protein acidic and rich in cysteine [16], human leukocyte antigen [31-33], mitochondrial complex I genes [34], and collagen type IV, alpha 3 and collagen type IV, alpha four [35]. In recent genome-wide association studies, hepatocyte growth factor [36] and 2q21.3 including RAB3 GTPase activating protein subunit 1 (catalytic) [37] were reported to be associated with keratoconus. However, studies in other populations failed to replicate previously reported associations for some of these candidate genes [16,17,21,22,38], and for other candidates, replication studies have not been reported.

Interleukin (IL)-1 is a proinflammatory cytokine that induces the production of cytokines and chemokines and plays an important role in inflammatory processes. IL-1 is involved in various cellular activities, including cell proliferation, differentiation, and apoptosis. The IL-1 family consists of two proinflammatory cytokines (IL-1α and IL-1β) and the IL-1 receptor antagonist (IL-1Ra). These proteins are encoded by *IL1A*, *IL1B*, and *IL1RN*, respectively, which comprise a cluster spanning 360 kb on chromosome 2q14. Recently, Kim et al. [39] investigated the association of polymorphisms in *IL1A*, *IL1B*, and *IL1RN* with keratoconus in a Korean population and reported that *IL1B* promoter polymorphisms rs1143627 (−31 T>C) and rs16944 (−511 C>T) are significantly associated with an increased risk of keratoconus. The study also suggested that the heterozygous genotype of the *IL1A* intronic polymorphism rs2071376 (+376 C>A) is associated with a decreased risk of the disease. However, no replication study has been performed in other ethnic populations. The aim of the present study was to investigate whether genetic polymorphisms in the *IL1A*-*IL1B* region are associated with the risk of keratoconus in Japanese patients.

## Methods

We recruited 169 unrelated Japanese patients with keratoconus and 390 unrelated healthy Japanese controls at Yokohama City University, Ryogoku Eye Clinic, Tokyo Dental College Ichikawa General Hospital, and Yamaguchi University in Japan. The diagnosis of keratoconus was based on slit-lamp biomicroscopic findings in one or both eyes, including corneal stromal thinning, Fleischer ring, Vogt's striae, or Munson’s sign by cornea specialists at each institute. The diagnosis was also based on characteristic patterns on video keratography: inferior corneal steepening, inferocentral corneal thinning, or an asymmetric bowtie with skewed radial axis. The mean age of the patients was 33.8±9.6 years; 76.2% of patients were male, and 23.8% were female. Controls were healthy volunteers from a geographic region similar to that for the keratoconus patients, and the control population was comparable in age and sex to the patients (mean age, 33.4±9.4 years; 74.6% male and 25.4% female). According to the Online Mendelian Inheritance in Man database [40], several diseases have been associated with the *IL1A* and/or *IL1B* polymorphisms: periodontitis, Alzheimer disease, osteomyelitis, end stage renal disease, gastric cancer, inflammatory bowel disease, Parkinson disease, and diabetic nephropathy. These diseases were not included in the patient and control groups. This study was approved by the ethics committee of each participating institute and complied with the guidelines of the Declaration of Helsinki. All study details were explained to all patients and controls before consent was obtained for genetic screening.

Genomic DNA was extracted from peripheral blood samples using the QIAamp DNA Blood Mini Kit (Qiagen, Hilden, Germany). Procedures were performed under standardized conditions to prevent variation in DNA quality. We evaluated three single nucleotide polymorphisms (SNPs) reportedly associated with keratoconus in a Korean population: rs2071376 in *IL1A*, and rs1143627 and rs16944 in *IL1B*. Genotyping of all SNPs was performed using the TaqMan 5′ exonuclease assay with validated TaqMan primer-probe sets supplied by Applied Biosystems (Foster City, CA). Polymerase chain reaction (PCR) was performed in a reaction mixture with a total volume of 10 μl containing 1X TaqMan Universal PCR Master Mix (Applied Biosystems), 24 nm of each primer-probe set, and 3 ng genomic DNA. The PCR conditions were as follows: 95 °C for 10 min, followed by 40 cycles of denaturation at 92 °C for 15 s and annealing/extension at 60 °C for 1 min. The probe fluorescence signal was detected using the StepOnePlus Real-Time PCR System (Applied Biosystems).

Allele and genotype frequencies were estimated with direct counting. Haplotype frequencies, Hardy–Weinberg equilibrium (HWE), and linkage disequilibrium (LD) were assessed using Haploview 4.1 software [41]. Differences in allele, genotype, and haplotype frequencies between cases and controls were assessed with χ^2^. To obtain a measure of significance corrected for multiple testing bias, we used the Bonferroni method. A corrected p (pc) value of <0.05 was considered statistically significant.

## Results

### Hardy–Weinberg equilibrium tests and haplotype block

**Figure 1:**
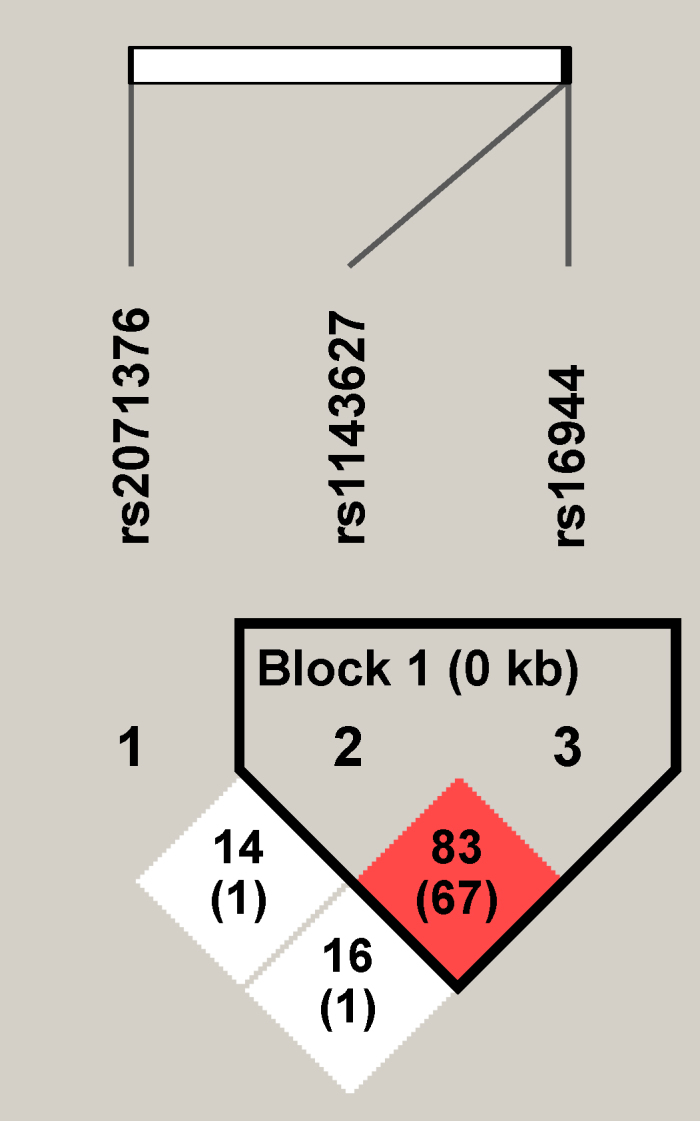
Linkage disequilibrium plot of three interleukin 1 alpha to interleukin 1 beta single nucleotide polymorphisms in 559 study participants. The D’ value and r^2^ value (in parentheses) corresponding to each single nucleotide polymorphism (SNP) pair are expressed as a percentage and shown within the respective square. Red represents a high-pairwise D' value.

The three SNPs were in HWE among the cases and the controls. Figure 1 shows the strength of LD for the three SNPs in all 559 participants. *IL1B* promoter SNPs rs1143627 and rs16944 were located in one haplotype block, and the magnitude of LD between the SNPs was extremely high (D’=0.83, r^2^=0.67). *IL1A* SNP rs2071376 was not linked with the *IL1B* SNPs.

### Allele and genotype frequencies

Table 1 provides the genomic locations and allele frequencies for the three SNPs. In *IL1B*, the frequency of the T allele of rs1143627 was significantly increased in the cases compared to the controls (60.7% versus 52.7%, p=0.014, pc=0.043, odds ratio [OR]=1.38). The C allele frequency of rs16944 was also increased in the cases compared to the controls (60.7% versus 53.7%, p=0.033, OR=1.33), although this increase did not reach statistical significance after the Bonferroni correction was performed (pc=0.098). Conversely, the frequency of the A allele of rs2071376 in *IL1A* was slightly higher among the cases compared to the controls, but no significant association was observed (27.7% versus 24.9%, p=0.337, OR=1.15).

**Table 1:** Allele frequencies of three SNPs in *IL1A* and *IL1B*

SNPs		Chr.	Position (Build 37.1)	Gene	Gene location	Alleles (1>2)	Risk allele	Risk Allele Frequency, %		*P*	*P*c	OR (95%CI)
								Cases (n=169)	Controls (n=390)			
rs2071376	(+376 C>A)	2	113,535,395	*IL1A*	Intron	C>A	A	27.7	24.9	0.337		1.15 (0.86–1.54)
rs1143627	(−31 T>C)	2	113,594,387	*IL1B*	Promoter	T>C	T	60.7	52.7	0.014	0.043	1.38 (1.07–1.79)
rs16944	(−511 C>T)	2	113,594,867	*IL1B*	Promoter	C>T	C	60.7	53.7	0.033	0.098	1.33 (1.02–1.72)

1, major allele; 2, minor allele; OR, odds ratio; CI, confidence interval. Allele frequency differences were tested using 2×2 contingency tables.

The genotype frequencies of the three SNPs are shown in Table 2. Individuals carrying two copies of the disease-risk allele of rs1143627 and rs16944 had 1.52- and 1.45-fold increased risks of keratoconus, respectively, and a marginal significant p value of 0.033 was observed for the TT genotype of rs1143627 (not significant after correction). For rs2071376 in *IL1A*, the AA genotype had a 1.76-fold increased risk of keratoconus, but it was not significant. In addition, the AC genotype of rs2071376 reported in a previous study [39] was not associated with the disease.

**Table 2:** Genotype frequencies of three SNPs in *IL1A* and *IL1B*

SNPs		Alleles (1>2)	Genotypes	Frequency, %		*P*	*P*c	OR (95%CI)
				Cases (n=169)	Controls (n=390)			
rs2071376	(+376 C>A)	C>A	AA	8.3	4.9	0.117		1.76 (0.86–3.60)
			AC	38.7	40.1	0.763		0.94 (0.65–1.37)
			CC	53.0	55.0	0.654		0.92 (0.64–1.32)
rs1143627	(−31 T>C)	T>C	CC	15.4	22.2	0.064		0.64 (0.39–1.03)
			CT	47.9	50.1	0.633		0.92 (0.64–1.31)
			TT	36.7	27.6	0.033	0.099	1.52 (1.03–2.23)
rs16944	(−511 C>T)	T>C	CC	36.7	28.6	0.058		1.45 (0.99–2.12)
			CT	47.9	50.3	0.613		0.91 (0.63–1.31)
			TT	15.4	21.1	0.115		0.68 (0.42–1.10)

1, major allele; 2, minor allele; OR, odds ratio; CI, confidence interval. Individual genotype frequency differences were tested using 2×2 contingency tables for each genotype by combining the remaining genotypes into one category.

### Haplotype analysis

A haplotype consisting of rs1143627 and rs16944 was observed (Table 3). The frequency of haplotype TC created by the risk alleles was significantly increased in cases compared to controls (60.4% versus 47.1%, p=4.0×10^−5^, pc=1.6×10^−4^, OR=1.72). In contrast, the frequencies of haplotype CC and TT were decreased in the cases compared to the controls with OR=0.03 (CC: 0.2% versus 6.7%, p=2.9× 10^−6^, pc=1.2×10^−5^; TT: 0.2% versus 5.7%, p=2.1× 10^−5^, pc=8.4×10^−5^).

**Table 3:** Haplotype frequencies of rs1143627 and rs16944 in *IL1B*

Haplotypes	Frequency, %		*P*	*P*c	OR (95%CI)
	Cases (n=169)	Controls (n=390)			
rs1143627-rs16944					
TC	60.4	47.1	4.0×10^−5^	1.6×10^−4^	1.72 (1.33–2.23)
CT	39.1	40.6	0.643		0.94 (0.72–1.22)
CC	0.2	6.7	2.9×10^−6^	1.2×10^−5^	0.03 (0.003–0.31)
TT	0.2	5.7	2.1×10^−5^	8.4×10^−5^	0.03 (0.003–0.37)

1, major allele; 2, minor allele; OR, odds ratio; CI, confidence interval. Individual haplotype frequency differences were tested using 2×2 contingency tables for each haplotype by combining the remaining haplotypes into one category.

## Discussion

The aim of the present study was to assess whether polymorphisms in the *IL1A*-*IL1B* region affect the development of keratoconus in a Japanese population. Therefore, we genotyped three polymorphisms in the region. Here, we report a significant association between *IL1B* promoter polymorphism rs1143627 and keratoconus in Japanese patients, suggesting that the *IL1B* promoter polymorphisms contribute to the risk of keratoconus in Japanese and Korean populations.

The SNPs rs1143627 is at the −31 position in the promoter region of *IL1B*. As promoter sequences are potential sources of polymorphisms affecting gene expression, rs1143627 may play critical roles in *IL1B* gene expression, which may contribute to the risk of keratoconus. This hypothesis is supported by previous studies showing that *IL1B* promoter polymorphisms, especially the T allele of rs1143627 in the TATA box, can enhance the expression of *IL1B* [42-45]. The IL1-B protein has been detected in human corneal epithelial, stromal fibroblast, and endothelial cells [46], and expression of this protein has reportedly been enhanced in keratoconus corneas compared to normal corneas [47]. In keratoconus, keratocyte apoptosis has been suggested to contribute to the corneal thinning process [48-50], which suggests that the enhanced *IL1B* expression caused by the promoter polymorphism, rs1143627, can induce the overexpression of IL1-B protein, resulting in the increased corneal apoptotic activity observed in patients with keratoconus. None of the previous linkage studies of keratoconus has ever detected 2q14 locus harboring *IL1B* [8]. Linkage studies have less power than case-control association studies to detect small or modest genetic effects [51]. *IL1B* polymorphisms have conferred modest risk for keratoconus (e.g., OR=1.3–1.5) in the present and previous studies [39], and therefore, the previous linkage studies may not have been able to find 2q14.

The previous study showed that only the heterozygous genotype of *IL1A* SNP rs2071376 was associated with keratoconus in the Korean population, whereas the allele frequencies were not significantly different between the patients and the controls [39]. In the present study, there was no association between keratoconus and any allele or genotype of rs2071376 in the Japanese population. Since rs2071376 was not linked with the *IL1B* SNPs, rs1143627 and rs16944, in the Japanese and Korean populations [39], the possibility that *IL1A* SNP rs2071376 is primarily associated with the development of keratoconus is low.

Keratoconus is genetically heterogeneous, and detecting susceptibility genes could provide useful information regarding the etiology of this poorly understood disease. In the present study, we found

that the *IL1B* promoter polymorphism, rs1143627, is associated with keratoconus in the Japanese population. This finding is in line with a previous study in a Korean population, suggesting that the *IL1B* promoter polymorphism is an important risk factor for susceptibility to keratoconus. Further genetic and functional studies are needed to clarify the contribution of the *IL1B* promoter region to the development of keratoconus.
